# Genome-wide profiles of methylation, microRNAs, and gene expression in chemoresistant breast cancer

**DOI:** 10.1038/srep24706

**Published:** 2016-04-20

**Authors:** Dong-Xu He, Feng Gu, Fei Gao, Jun-jun Hao, Desheng Gong, Xiao-Ting Gu, Ai-Qin Mao, Jian Jin, Li Fu, Xin Ma

**Affiliations:** 1 National Engineering Laboratory for Cereal Fermentation Technology, Jiangnan University, Wuxi 214122, China; 2 Department of Breast Cancer Pathology and Research Laboratory, State Key Laboratory of Breast Cancer Research, Cancer Institute and Hospital, Tianjin Medical University, Tianjin 300060, PR China; 3 Agricultural Genomes Institute at Shenzhen, Chinese Academy of Agricultural Sciences, Shenzhen 518120, China; 4 State Key Lab of Genetic Resources and Evolution, Kunming Institute of Zoology, Chinese Academy of Sciences, Kunming, Yunnan 650223, China; 5 Department of Cellular and Molecular Pharmacology, School of Pharmaceutical Sciences, Jiangnan University, Wuxi 214122, China

## Abstract

Cancer chemoresistance is regulated by complex genetic and epigenetic networks. In this study, the features of gene expression, methylation, and microRNA (miRNA) expression were investigated with high-throughput sequencing in human breast cancer MCF-7 cells resistant to adriamycin (MCF-7/ADM) and paclitaxel (MCF-7/PTX). We found that: ① both of the chemoresistant cell lines had similar, massive changes in gene expression, methylation, and miRNA expression *versus* chemosensitive controls. ② Pairwise integration of the data highlighted sets of genes that were regulated by either methylation or miRNAs, and sets of miRNAs whose expression was controlled by DNA methylation in chemoresistant cells. ③ By combining the three sets of high-throughput data, we obtained a list of genes whose expression was regulated by both methylation and miRNAs in chemoresistant cells; ④ Expression of these genes was then validated in clinical breast cancer samples to generate a 17-gene signature that showed good predictive and prognostic power in triple-negative breast cancer patients receiving anthracycline-taxane-based neoadjuvant chemotherapy. In conclusion, our results have generated a new workflow for the integrated analysis of the effects of miRNAs and methylation on gene expression during the development of chemoresistance.

Over the past decades, the survival rates of patients with breast cancer have markedly increased, partly due to improvements in chemotherapy. Regimens based on anthracyclines (doxorubicin, daunomycin, and epirubicin) and taxanes (paclitaxel and docetaxel) are the most frequently used combination therapy for breast cancers^1^. However, chemoresistance is the main cause of chemotherapeutic failure and leads to suboptimal response rates. To date, several critical mechanisms have been found to contribute to chemoresistance in breast cancers. For example, over-expression of p-glycoprotein^2^,^3^ and glutathione-s transferase^4^ have been shown to contribute to chemoresistance. Moreover, chemoresistance is regulated by the epithelial mesenchymal transition (EMT) pathway^5^,^6^. Cancerous epithelial cells lose their polarity and cell-cell adhesion, and gain high motility during the EMT *via* pathways including but not limited to TGF-beta, Wnt/beta-catenin, Notch, and Hedgehog. Mobile cancer cells then move away from unfavorable environments targeted by chemotherapeutic agents and subsequently cause therapy insensitivity and metastasis. In addition, pathways in EMT enable cancer cells to proliferate quickly and resist to apoptotic signals, so that the cancer cells become less sensitive to the cytotoxicity from chemotherapy.

Nevertheless, chemoresistance is mediated by a wide range of poorly-understood genomic and epigenetic networks, and these mechanisms have not been systematically analyzed in chemoresistant breast cancers. DNA methylation and microRNA (miRNA) silencing are known to be important in chemoresistance^7^,^8^; they either suppress or activate a substantial proportion of genes at the pre- and post-transcriptional levels, respectively. Because chemoresistance involves multiple interacting factors, it is not sufficient to investigate the methylation and miRNA regulation of a single factor. Therefore, understanding the network of methylation and miRNA regulation and their relationship in chemoresistant cancer cells would provide valuable information during the acquisition of chemoresistance.

In this study, high-throughput reduced representation bisulfite sequencing (RRBS), and RNA sequencing (RNA-Seq) of the transcriptome profiles of coding messenger RNAs and non-coding small RNAs in chemoresistant and chemosensitive breast cancer cell lines were analyzed in-depth in order to identify the dysregulated targets in chemoresistant cells, and to develop a signature to predict chemoresistance and prognosis. The workflow of this study is presented in Fig. S1.

## Results

### Generation of comparative profiles of DNA methylation, and mRNA and miRNA expression in chemoresistant and chemosensitive MCF-7 cell lines

The genome-wide DNA methylation patterns of the adriamycin (ADM)- and paclitaxel (PTX)-resistant human breast cancer cells (MCF-7/ADM and MCF-7/PTX), as well as their drug-sensitive parental control MCF-7/WT cells^9^, were analyzed by RRBS sequencing. RRBS is widely used to measure the DNA methylation of high-CG regions at single base-pair resolution. A total of 141 million pair-end 50-bp reads were obtained for each cell line. By allowing less than two mismatches, 93.56% of the clean reads were mapped back to the hg19 human reference genome, reaching a unique mapping rate of 76.22% and a >20× read depth (Table S1a). Nearly 91% of promoters and 94% of the CpG islands were detected (Table S1b). In agreement with previous reports^10^, the methylation levels of coding sequences, introns, and 3′-untranslated regions (UTRs) were generally high, while the 5′-UTRs and promoters were hypomethylated (Fig. S2a). As the majority of the methylated cytosines (~98%) were in a CpG context (Fig. S2b), we performed the subsequent analyses of a total of 2.88 million CpG sites with >4× coverage depths that were commonly reached in the three cell lines.

**Figure 1:**
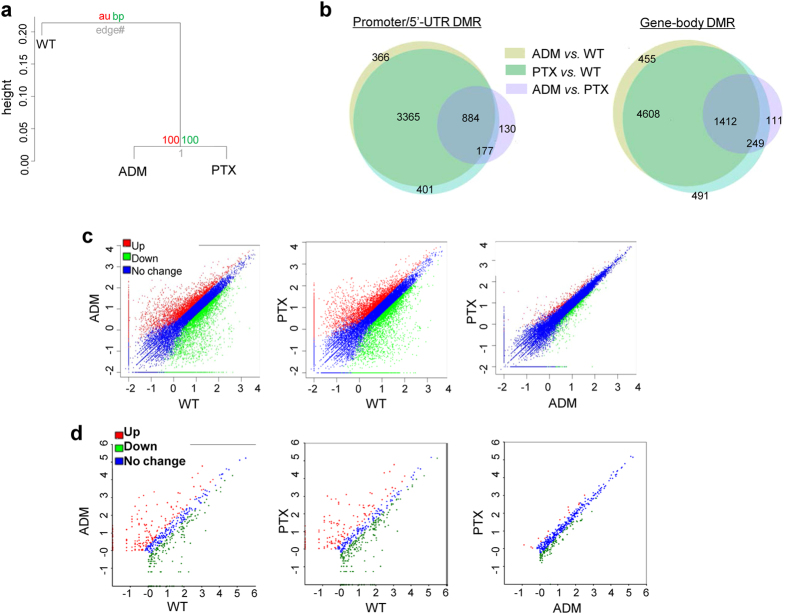
Generation of comparative profiles of DNA methylation, mRNA and miRNA expression. (**a)** Hierarchical clustering of the methylation status in MCF-7/ADM, /PTX, and /WT cells. **(b)** Venn diagrams of the DMRs of the three comparison groups. **(c**,**d)** Scatter-plots of the differentially-expressed mRNAs (**c**) and miRNAs (**d**). Fold-changes of mRNAs and miRNAs are shown on the X or Y axis as Log (fold-change value).

Hierarchical clustering analysis of their methylation status showed that MCF-7/ADM and MCF-7/PTX cells fell into the same cluster, but were separate from the parental control MCF-7/WT cells (Fig. 1a). Pair-wise comparisons were performed among the three cell lines to identify the differentially-methylated regions (DMRs) with statistical significance (median β differences >0.20, p < 0.01), and DMRs were then annotated to genes based on their genomic positions. The majority of the DMRs were allocated to gene-body regions and intergenic regions, while <15% were in promoters and the 5′-UTR (Fig. S2c). Furthermore, most of the DMRs in genes overlapped in the two chemoresistant groups compared with their chemosensitive comparison groups (Fig. 1b). Therefore, these data suggested that the methylation pattern is similar in the two chemoresistant cell lines.

Furthermore, we performed RNA-Seq on coding mRNAs (mRNA-Seq) and non-coding small RNAs (sRNA-seq) in the three MCF-7 cell lines (Table S1c and d). More than 7 and 16 million clean single-end 50-bp reads were obtained in mRNA-Seq and sRNA-Seq, reaching high coverage of ~75% and 70% of the annotated coding genes and sRNAs in the human genome, respectively. Since miRNAs are one of the most-studied sRNAs in oncology, we focused on miRNA expression in the subsequent analyses. We then performed pair-wise comparisons of mRNA and miRNA expression levels with the same criteria for a significant difference (FDR ≤0.001 and |log_2_Chemoresistant/WT| ≥1). Consistent with the genome-wide DNA methylation status, scatter-plot analyses showed that a large number of genes significantly differed between chemoresistant (MCF-7/ADM and /PTX) cells and their parental control cell line MCF-7/WT, both for mRNA and miRNA expression. However, the expression patterns were quite similar in the two chemoresistant cell lines (Fig. 1c,d).

Taken together, the three sets of high-throughput sequencing results indicated the common molecular changes, both in DNA methylation and gene expression, in breast cancer cells with resistance to different chemotherapeutic drugs.

### Cell motility and EMT genes are enriched in combined analyses of differential DNA methylation and mRNA expression

We then cross-matched the DMR-containing genes from RRBS with differentially-expressed genes from mRNA-Seq. Since the two chemoresistant cell-lines again showed great overlap of matched genes, we focused on these overlapped genes in the subsequent analyses in order to find common pathways in chemoresistant cancer cells (Table S2).

**Figure 2:**
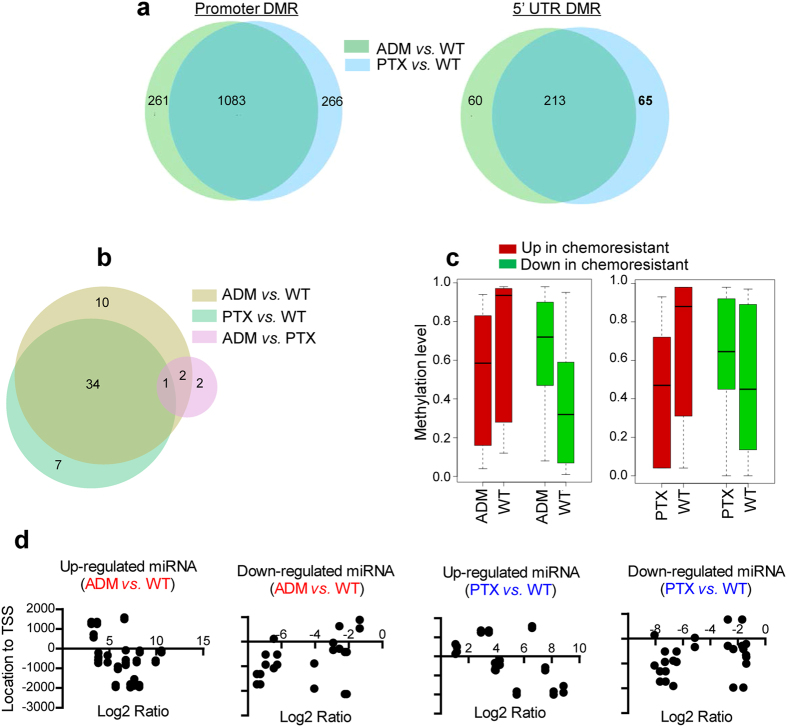
Cross-matching of sequencing data. (**a)** Venn diagrams of the differentially-expressed genes cross-matched with the DMRs in their promoter and 5′UTR regions in the three comparison groups. Genes were chosen when their expression changes were negatively associated with changes in the DMRs in chemoresistant cells *versus* MCF-7/WT cells. **(b)** Venn diagrams of the differentially-expressed miRNAs cross-matched with DMRs around the miRNA coding sequence in the three comparison groups. **(c)** Up- or down-regulated miRNAs were integrated with the overall methylation level when comparing resistant with sensitive cells. Red bars, overall methylation levels (Y axis) of up-regulated miRNAs in MCF-7/ADM or /PTX *versus* /WT cells; green bars, overall methylation levels of down-regulated miRNAs in MCF-7/ADM or /PTX *versus* /WT cells. **(d)** Locations of the DMRs relative to the TSS of each miRNA. X axis, Log_2_ values of DMRs (resistant *versus* sensitive); Y axis, their locations relative to the TSS.

Consistent with the widely-accepted mechanism of methylation^11^, the expression of 1083 and 213 genes in both MCF-7/ADM and /PTX cells was negatively correlated with the levels of promoter and 5′UTR methylation, respectively, *versus* MCF-7/WT cells (Fig. 2a). This suggested that methylation plays a role in gene-silencing during the acquisition of chemoresistance. The mechanism by which methylation regulates expression in the gene-body is complicated, but more than half of these regions were positively correlated with gene expression as previously suggested (Table S2)^12^.

To better understand the potential effects of methylation-related changes in gene expression on cellular functions, we performed Pathway Commons (PC) and Kyoto Encyclopedia of Genes and Genomes (KEGG) analyses using a web-based gene set analysis toolkit (WebGestalt; http://bioinfo.vanderbilt.edu/webgestalt/). The results showed enrichment in cancer and metabolic pathways (Table S3), suggesting that the challenge of chemotherapeutic drugs and the development of chemoresistance trigger pathways relevant to tumor metabolism. More interestingly, cell mobility and cellular surface interaction pathways (extracellular matrix-receptor interaction, cell adhesion molecules, MAPK signaling pathway, and focal adhesion in KEGG, along with integrin family cell-surface interactions in PC) were highly enriched. These pathways are directly associated with cell motility and include key proteins in the EMT signaling pathways^5^,^6^.

Therefore, the above observations led us to hypothesize that change in motility that is a key feature of EMT, as well as the EMT itself, are key targets of regulation by methylation during the acquisition of chemoresistance. To test our hypothesis, we analyzed the motility and mesenchymal features of chemoresistant cells. Migration was increased in both MCF-7/ADM and MCF-7/PTX cells, indicating increased cell motility (Fig. S3a). Meanwhile, the expression and plasma membrane distribution of E-cadherin (expressed by *CDH1*) decreased and those of N-cadherin (expressed by *CDH2*), vimentin, snai1, and twist increased in both of the chemoresistant cell lines (Fig. S3b–d), suggesting that these cells had undergone the EMT process. These data verified and supported the results and conclusions of high-throughput analysis.

We also used real-time PCR to analyze the expression of other EMT-related genes and miRNAs discussed below (Fig. S4); the results confirmed the onset of the EMT. Also, most of the real-time PCR results are consistent with high-throughput sequencing, suggesting the accuracy of the high-throughput sequencing in determining the expression change of genes.

### Integration of miRNA expression with mRNA expression and DNA methylation respectively

We next integrated the mRNA-Seq with the sRNA-Seq results. The theoretical targets of all of the significantly changed miRNAs were predicted by MirTarget2, miRecords, and miRanda software together. These predicted targets were then integrated with the mRNA-Seq data. Consequently, a group of miRNAs was found to overlap in the two chemoresistant cell lines (Table S4). First, the miRNA expression significantly changed; second, the expression of their theoretical mRNA targets significantly changed; and third, the expression of these miRNAs was inversely correlated with their target mRNA expression. These results thus provide a refined pool of miRNAs with major implications for chemoresistance. Based on this, we found that miR-489 and its target *Smad3* regulate chemoresistance *via* the EMT pathway^13^, suggesting such analysis could simplify the way to find important miRNAs and its targets regulating chemoresistance, and again indicating the important role of the EMT in the regulation of chemoresistance.

We investigated several of the motility- and EMT-related miRNAs listed in Table S4 based on our findings that motility and the EMT were primarily regulated in chemoresistant cells. We found that the expression of all of these miRNAs changed dramatically. When the dysregulation of these miRNAs in MCF-7/ADM or /PTX cells was inhibited with miRNA inhibitors or mimics, the chemoresistance to ADM and PTX was decreased as determined by the IC_50_s (half-maximal inhibitory concentrations) of the drugs (Table S4), suggesting that the motility and EMT processes that are regulated by miRNAs also play important roles in chemoresistance.

The sRNA-Seq results were then integrated with the RRBS results. Recent studies have suggested that miRNA expression can be regulated by methylation of the promoter region^14^. Thirty-four dysregulated miRNAs were surrounded by differentially-methylated regions near the transcription start site (TSS) of the miRNA coding sequence (±2000 bp from the TSS) in both MCF-7/ADM and /PTX cells *versus* MCF-7/WT cells (Table S5, Fig. 2b). Generally, the expression of these miRNAs was negatively correlated with the overall methylation around their TSSs. miRNAs up-regulated in MCF-7/ADM and /PTX cells showed an overall decrease in methylation level around their TSSs *versus* MCF-7/WT cells, and *vice versa* (Fig. 2c). Investigation of individual locations showed that some of the methylation was situated in the -2000 region of the TSS, which is a theoretical promoter region of miRNAs (Fig. 2d)^15^, suggesting that their expression might be regulated by promoter methylation. For example, among the EMT/motility-related miRNAs listed in Table S4, miRNA-200c and miRNA-34a have been reported to be regulated by methylation in malignant cancer cells^16^,^17^, and our results further indicated that regulation by methylation occurs in chemoresistant cancer cells. In addition, we found for the first time that miR-106a-5p and miRNA-375 may be regulated by methylation in chemoresistant cells. Based on these data, we confirmed that down-regulation of miR-149-5p is associated with methylation and chemoresistance^18^, which indicate the accuracy of this step of integration.

### Combined analysis of DNA methylation, and mRNA and miRNA gene expression

**Figure 3:**
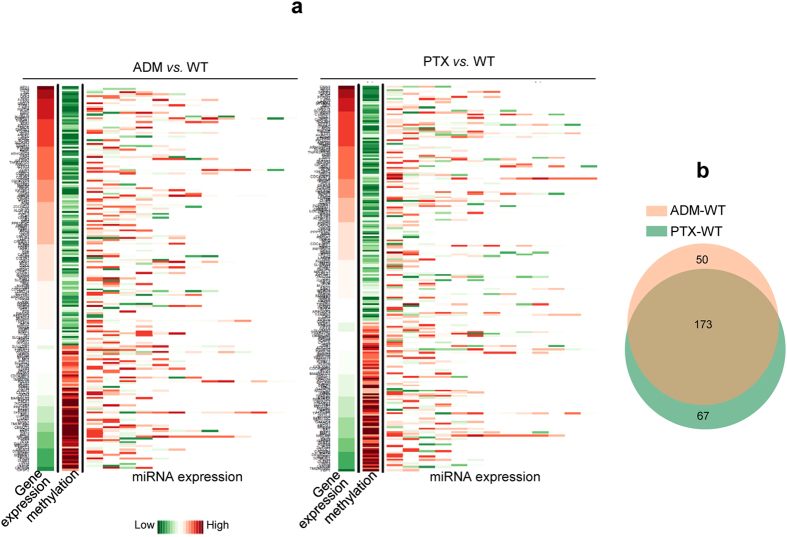
Integration of RRBS, mRNA-Seq, and sRNA-Seq. (**a)** Genes whose expression was negatively associated with promoter methylation were used in the integration. Left lanes of the heat map are gene-expression values in MCF-7/ADM (left panel) and /PTX (right panel) *versus* /WT cells; middle lanes are the DMR values for each gene; right lanes are the expression of predicted miRNAs targeting each gene. **(b)** Venn diagrams of genes both regulated by methylation and miRNA in resistant *versus* sensitive comparison groups.

In the final step of high-throughput data integration, we investigated both pre- and post-transcriptional control of single genes during the acquisition of chemoresistance by combining mRNA-Seq, RRBS, and sRNA-Seq data (Table S6). In the MCF-7/ADM *versus* WT and MCF-7/PTX *versus* WT comparison groups, 223 and 240 genes with considerable overlaps were generated, respectively (Fig. 3a,b). The expression of genes in these lists was negatively correlated with promoter methylation (gene-body methylation was excluded from this part of the analysis because its regulatory mechanism is more complicated than promoter methylation). Meanwhile, all of the miRNAs predicted to target certain genes were listed, and the expression of each gene was negatively regulated by at least one miRNA.

The genes doubly-regulated by methylation and miRNA were then categorized by KEGG assay (Table S7). The largest categories fell again into cell motility- and EMT-related groups, such as cell adhesion molecules (KEGG 04514), focal adhesion (KEGG 04510), and extracellular matrix-receptor interaction (KEGG 4512), suggesting that the EMT and the related motility are the main targets of methylation and miRNA regulation in chemoresistant cells.

**Figure 4:**
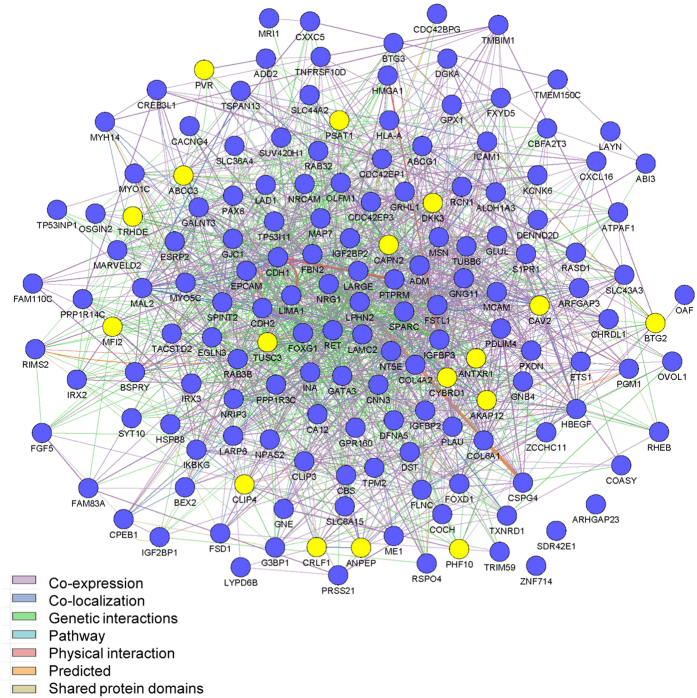
Molecular interactions of genes. The interactions were analyzed by Cytoscape_3.2.0 plugin GeneMANIAThe query genes were marked blue, and the genes in 17-gene signature were marked yellow.

In order to better understand the molecular interaction of these genes, the interaction network of the genes were analyzed by Cytoscape 3.2.0^19^ plugin GeneMANIA^20^, where gene interactions were collected from interaction datasets BioGRID^21^. In such analysis, two genes are functionally linked if the effects of perturbing one gene were found to be modified by perturbations to a second gene. As data showed (Fig. 4), these genes showed extensive co-expression, genetic interactions and physical interactions, suggesting they are densely connected to modulate chemoresistance development. Among them, EMT regulators, such as CDH1, EPCAM, CDH2^5^,^6^ were in the central place of the network, suggesting again the importance of EMT pathway. Furthermore, several genes such as CAPN2, TUSC3 and CYBRD1, played as central factors in the networks and validated in clinical samples as we demonstrated later. Therefore, their molecular mechanisms are worth exploring.

### Generating and verifying the chemoresistance signature

Clinically, chemoresistance can only be detected during the middle-to-late stages of chemotherapy, and there are no diagnostic tests to recognize resistance before treatment. To confront this difficulty, genes that were both targeted by methylation and miRNA from the last step of integration (termed double-regulated genes) were validated in breast cancer samples from 27 patients receiving neoadjuvant taxane-anthracycline (TA)-based chemotherapy (Table S8). Thirteen patients were sensitive, as they responded to chemotherapy (complete response, CR), while 14 were resistant (progressive/stable disease after chemotherapy, PD/SD).

Immunohistochemical analysis of the chemoresistant and sensitive groups showed that the expression changes of most of the genes found in MCF-7 cells were more consistent with those from triple-negative breast cancer (TNBC) patients, than from all individuals regardless of their estrogen receptor (ER), progesterone receptor (PR) and human epidermal growth factor receptor 2 (Her2) status. This makes sense because the MCF-7/ADM and /PTX cells did not express ER, PR and Her2 (Fig. S5a), and loss of control of these receptor may making them resemble features of TNBC cell lines^22^,^23^,^24^. Seventeen genes were selected from the list of double-regulated genes, and these also greatly/significantly differed in the clinical pre-chemotherapeutic samples between chemoresistant and chemosensitive TNBC patients (Table 1 and +5b).

**Figure 5:**
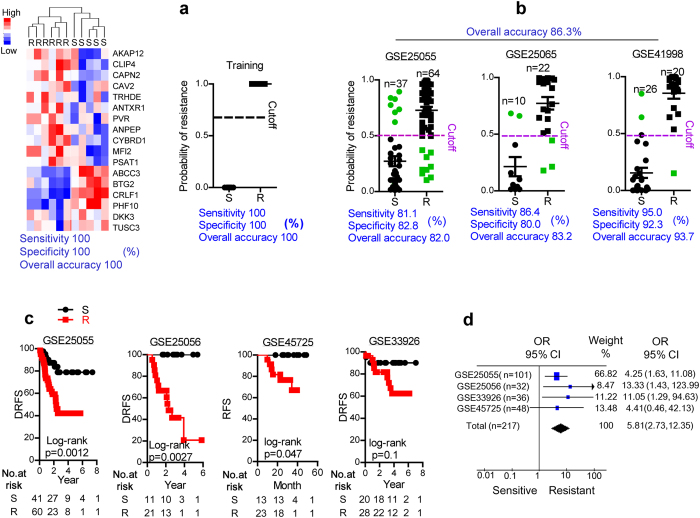
Clinical validation of the signature. (**a)** Validation of the signature in resistant (R) and sensitive (S) TNBC patients from the training cohort with hierarchical clustering (left panel) and Bayesian binary regression (right panel). **(b)** Validation of the signature in TNBC patients in the GSE25055 (left panel), 25065 (middle panel), and 41998 (right panel) databases by Bayesian binary regression. **(c)** Effectiveness of the signature in predicting the prognosis in TNBC patients from the GSE25055 (left panel), 25065 (2^nd^ left panel), 45725 (right panel), and 33926 (2^nd^ right panel) databases. Patients were grouped according to their potential chemoresponse predicted by the signature. **(d)** Forest plots of the performance of the chemoresistance signature in predicting clinical outcomes. Odds ratios (OR) are plotted as vertical colored bars for comparison. The length of the horizontal bars and the width of the diamonds correspond to 95% confidence intervals. The diamond represents the total OR of the signature in the total cases. The weight means the size of each dataset *versus* the total cases.

The discriminatory power of the signature was analyzed first in the training cohort formed by the TNBC samples we had collected. Hierarchical clustering^25^ and Bayesian binary regression^26^ were used as classifiers. The signature classified the chemoresistant and chemosensitive patients with an overall accuracy of 100% for hierarchical clustering and 100% for Bayesian binary regression (Fig. 5a), suggesting that the signature can discriminate between resistant and sensitive TNBC patients.

The 17-gene signature was then validated in three independent cohorts of TNBC breast cancer patients receiving TA-based chemotherapy. Affymetrix gene expression profiling of pre-chemotherapeutic TNBC samples from previous studies (NCBI Nos GSE 25055, 25065, and 41998)^27^,^28^ were used. Patients showing a pathologically complete response were defined as chemosensitive, and those with an extensive residual cancer burden (RCB-II to III) or residual disease were considered to be chemoresistant. By Bayesian binary regression analysis, the signature showed good discrimination of both resistant and sensitive patients (i.e., the sensitivity and specificity of the signature). The overall accuracy was as good as 86.3% (Fig. 5b).

Because a good chemoresponse often leads to a better recovery rate, we tested the prognostic effectiveness of the 17-gene signature. For GSE25055 and GSE25065, where both the chemoresponse and distant metastasis relapse-free survival (DRFS) information were provided, the TNBC patients were grouped according to the 17-gene signature. The Kaplan-Meier results showed that patients classified as chemoresistant showed a worse rate of DRFS than chemosensitive patients in both groups (Fig. 5c).

In the other two databases (GSE45725 and 33926), only information on relapse-free survival (RFS) or DRFS was provided, without the chemotherapeutic responses. Therefore, Bayesian binary regression could not be calculated because of the lack of predetermined data to train the regression process^29^. So, the k-nearest neighbor method^30^ was used to classify the patients *versus* the average gene expression in sensitive and resistant patients in GSE25055 and 41998 (GSE25065 was excluded because its TNBC sample size is small). Kaplan-Meier analysis was then applied to the candidate resistant and sensitive patients, those who were probably chemoresistant as determined by the 17-gene signature showed a lower rate of RFS or DRFS than chemosensitive patients (Fig. 5c).

Finally, the risk of death/relapse in chemoresistant patients was summarized by the odds ratios (ORs) in forest plots (Fig. 5d). The mean OR for each dataset, as well as the total OR for all datasets, were >1 for potentially chemoresistant individuals defined by the 17-gene signature, suggesting that the signature is able to discriminate the population of TNBC patients at high risk of death/relapse.

## Discussion

DNA methylation and miRNA dysregulation are critical and effective mechanisms underlying the development of chemoresistance. A few studies have reported methylation and miRNA profiling in cancers^31^,^32^, but our study is the first to provide an overview of epigenetic regulation at the combined levels of DNA methylation, miRNA expression and gene expression in breast cancer chemoresistance, and then systematically identify novel dysregulated targets in chemoresistant breast cancer cells. We shared a refined and detailed pool of chemoresistant regulation with hundreds of possible targets, and one can easily find the possible interplays between methylation, miRNA and gene expression in their own interest from this pool. Experimental validation of certain interplay with siRNAs, over-expression vectors, CRISPR-Cas9 and other genome engineering methods allow indentifying the key players in chemoresistance, and deciphering their underlining molecular mechanism would be another step to conquering the chemoresistance. Based on the pool, we have shown the relationship and mechanism of several miRNAs and methylation sites during development of chemoresistance and validated them in clinical samples^13^,^18^,^33^, suggesting the pool is precise and valuable, and we are expecting more important mechanisms could be found in future.

One of the most important findings in this study is the high degree of similarity of both methylation and miRNA regulation in two chemoresistant breast cancer cell lines. They were developed from the same parental line but confronted entirely different chemotherapeutic challenges. To date, numerous critical mechanisms have been found to contribute to chemoresistance of different drugs^2^,^3^,^4^,^5^, some studies have also identified common gene or protein sets that are common to chemoresistance based on high throughput data methods such as proteomics^34^ and affymetrix microarray^35^. Our study further extended their results by identifying and emphasizing common interplays between genes, miRNAs and methylations in response to different chemotherapeutic agents. Such similar responses indicate that gene methylation, miRNAs, and the pathways we identified may also be shared by chemoresistance to other types of agents. The EMT pathway and associated high motility were consistently highlighted throughout our analyses. Although there are other important pathways that can mediate chemoresistance^2^,^3^,^4^,^5^, the data pointed out cancer cells preferentially and extensively evoked EMT pathway during the development of chemoresistance rather than other pathways, probably because under the stress of different and complex chemotherapeutic drugs, enhancing the ability to ‘escape’ and ‘survive’ may be the easiest and universal choice for all types of cancer cells. Therefore, EMT/motility-blocking drugs might be more robust against chemoresistance than traditional cytotoxic or cytostatic drugs. Furthermore, although a number of previous studies have shown mechanisms of EMT in chemoresistance, but fewer studies explained regulatory mechanism of methylation, miRNA or methylation and miRNA together on EMT signaling pathways in breast cancer chemoresistance. This study then provides information about how the key modulators in EMT pathway are regulated by epigenetic factors. Among those, we have validated EMT transcriptional factor SMAD3 is regulated by miR-489 to develop chemoresistance in breast cancers^13^. Therefore, after more validations are performed in future, it is expected to understand how the epigenetic ‘bridge’ is constructed between genotype and phenotype of cancer cell in response to chemotherapeutic stimuli via EMT pathway.

Based on the combination of methylation, miRNA, and gene-expression profiling, a 17-gene signature was generated and validated in TNBC. We need to make it clear that although MCF-7/ADM and PTX did not express ER, PR and Her2, but they are not equivalent to TNBC cells, which is p53 mutated and PTEN deleted, while MCF-7 cells are p53 wildtype with PI3 kinase mutations^36^. However, in confront to stress from chemotherapeutic toxicity, the MCF-7 cells dramatically changed their expression profiles to evolve into more malignant and aggressive ones as we also demonstrated in previous studies^9^,^37^, loss of ER, PR and Her2 may be a way for them to mimic the important pathways in TNBC cells to survive more easily^22^,^23^,^24^. Therefore, the significantly changed 17 genes in our signature may also represent critical pathways in TNBC cells, and the claim was supported by the clinical validation.

We particularly point out that the signature was generated from pre-chemotherapeutic samples, so it can predict the responsiveness of breast cancer patients before treatment. If tumor cells of the patients showed gene-expression changes in the 17-gene signature, these patients are more likely resistant to subsequent chemotherapies. However, when the patients are pre-determined to be chemosensitive with the 17-gene signature, they also require careful design for chemotherapeutical regimes. Aside from functional gene expression changes, tumor microenvironment is another important determinant to chemoresponse. When the chemotherapeutical drugs are given intravenously, tumor regions distal to vasculature are less frequently exposed to lethal dose of chemotherapeutical agents. Consequently chemoresistance is observed even when these cells are sensitive to therapies^38^. To overcome the microenvironment-induced chemoresistance, sequential treatment is one option because it can remove tumor cells near blood vessels and expose cells in deep regions during the first course of treatment, thus enables drugs to penetrate deeper through tumor tissue during the second course of treatment^39^. In agreement, we found some patients predicted to be chemosensitive in GSE41998 (where chemoresponse during different courses of chemotherapies was provided) showed poor response during first course of anthracyclines-based treatment, but they finally gained a good outcome after the sequential taxol-based treatment.

Furthermore, although we explored the roles of methylation and miRNAs in chemoresistance, we did not include these two factors in the signature for practical reasons: they are neither commonly used nor easy to test during clinical diagnosis.

The significant changes in expression of these 17 genes were associated with methylation and the dysregulation of miRNAs, as well as showing high relevance to the EMT process. Seven genes were mediators of the EMT: ANPEP (CD13)^40^, ANTXR1^41^, BTG2^42^, CAV2^43^, MFI2^44^, PSAT1 (proliferation)^45^, and PVR^46^. For some of these, EMT-related functions have not yet been found in breast cancer; for example, MFI2 has only been shown to mediate the EMT in melanoma, so further exploration of their roles in the EMT and chemoresistance in breast cancer is worthwhile. The expression of three genes, AKAP12^47^, DKK3^48^, and TUSC3^49^, in chemoresistant MCF-7 cells and TNBC samples was not consistent with previous reports. This may be due to differences in the types of cells used, so whether they play different roles in chemoresistant TNBC cells needs further study. Furthermore, there have been no reports on the activity of CLIP4, CAPN2, CRLF1, CYBRD1, PHF10, and TRHDE in breast cancer or chemoresistance, thus making them new candidates for understanding breast cancer, the EMT, and chemoresistance. Finally, ABCC3 is thought to mediate chemoresistance^50^ but is down-regulated in chemoresistant TNBC cells. This may be due to the fact that the role of ABCC3 in chemoresistance varies dramatically among different chemotherapeutic agents and different types of cells^51^, and our study suggests a new action of ABCC3 to potentially inhibit chemoresistance. We then showed the 17 genes were extensively co-expressed, genetic interacted and physical interacted with each other directly or indirectly. Because the expression of 17 genes are significantly related with chemoresistance in clinical samples, so further study may be performed to validate these interactions and to identify the dominant player in this group of genes, which is likely important determinants and treatment target to chemoresistance. Our preliminary data analysis suggests CAPN2, TUSC3 and CYBRD1, which sit in the central location in the network, might be good candidates.

Because the chemoresistant MCF-7 cells were developed under continuous ADM and PTX challenge, we chose samples from patients undergoing TA-based chemotherapy for the clinical validation of this study. As a result, we only demonstrated the good predictive and prognostic power of the signature in a TA-based regime, so its utility for other chemotherapeutic regimes needs further validation. However, we did find indirect evidence that the 17-gene signature may be effective for different chemotherapeutic agents: in the two validating datasets with unknown chemotherapeutic regimes (probably unrelated to TA), the signature classified TNBC patients into potentially resistant and sensitive groups. Although we cannot provide direct evidence for the accuracy of this classification, a worse prognosis for the potentially chemoresistant patients indirectly indicates that the signature correctly classifies TA-unrelated responses because of the tight connection between chemoresponse and prognosis. Such good discriminative capacity of the signature may be because the MCF-7/ADM and /PTX cells show features of multi-drug resistance^9^, so the signature derived from them may involve mechanisms of resistance common to other agents. These mechanisms are more reflective of the natural history of cancers, i.e, to proliferate and survive, rather than response to a simple chemotherapy. Indeed, a number of previous studies have shown the prognostic correlation of expression of EMT and proliferation genes^52^,^53^,^54^. Furthermore, taxane and anthracycline are commonly-used and effective agents, and their complementary mechanisms are specifically beneficial for TNBC^55^. Therefore, if TNBC cells show evident resistance to the TA-based regime, they are more likely to have already developed a mature mechanism of resistance to toxic drugs and be more malignant, so the signature could provide information about their response to commonly-used chemotherapeutic agents, and predict the prognosis of TNBC patients.

Taken together, our analysis not only provides a systematic means of integrating multiple levels of transcriptional regulation in chemoresistant cancer cells, but also presents several markers of cancer prognosis, therapeutic targets, and predictors of chemotherapeutic efficiency.

## Materials and Methods

Additional methods are described in supplemental information Materials and Methods.

### Cell culture

MCF-7/WT human breast cancer cells were obtained from the ATCC (HTB-22) in 2011. The adriamycin (ADM)- and paclitaxel (PTX)-resistant cells (MCF-7/ADM and MCF-7/PTX) were derived as previously described^4^ by treating MCF-7 cells with stepwise increasing concentrations of ADM and PTX over 8 months. These MCF-7 cells were cultured in RPMI supplemented with 10% FBS, 100 μg/mL penicillin, and 100 U/mL streptomycin. No authentication was done for these cell lines.

### Data deposition

The high-throughput data have been submitted to GEO (GSE68815).

### Combined analysis and functional enrichment

To compare the DNA methylation data with the miRNA expression levels, for each differentially-expressed miRNA we studied the CpGs within a 2000-bp window around the transcription start site. Several miRNA target databases were used for retrieving the known miRNA-targeted genes: MirTarget2 (http://mirdb.org/miRDB/), miRecords (http://mirecords.biolead.org), and miRanda (http://www.microrna.org/microrna/getDownloads.do). Only targets predicted in at least 2 of these databases were included in the subsequent analysis. WebGestalt (http://bioinfo.vanderbilt.edu/webgestalt/) was used for Gene Ontology and Pathway Enrichment analysis^42^.

### Ethics statement

This study was reviewed and approved by the Ethic Committee of Tianjin Medical University Cancer Institute & Hospital (Ethical approval number: bc201505). All procedures were carried out in accordance with approved guidelines. Each patient involved in this study provided written informed consent.

## Additional Information

**How to cite this article**: He, D.-X. *et al.* Genome-wide profiles of methylation, microRNAs, and gene expression in chemoresistant breast cancer. *Sci. Rep.* **6**, 24706; doi: 10.1038/srep24706 (2016).

## Supplementary Material

Supplementary Information

Supplementary Table

## Figures and Tables

**Table 1:** 17-gene signature.

Genes	*p* value	Down/up in resist	Genes	*p* value	Down/up in resist	Genes	*p* value	Down/up in resist
ABCC3	0.036	Down	CAV2	0.047	Up	PHF10	0.070	Down
AKAP12	0.080	Up	CLIP4	0.071	Up	PSAT1	0.010	Up
ANPEP	0.090	Up	CRLF1	0.004	Down	PVR	0.038	Up
ANTXR1	0.039	Up	CYBRD1	0.029	Up	TRHDE	0.094	Up
BTG2	0.089	Down	DKK3	0.076	Up	TUSC3	0.027	Up
CAPN2	0.032	Up	MFI2	0.046	Up			
