# Supplementary material for: Genome-wide profiles of methylation, microRNAs, and gene expression in chemoresistant breast cancer
Source: Sci Rep. 2016 Apr 20;6:24706. doi: 10.1038/srep24706 (PMC4837395; doi:10.1038/srep24706)
Supplement: Supplementary Information [file srep24706-s1.doc]

**Supplemental information**

Genome-wide profiles of methylation, microRNAs, and gene expression in chemoresistant breast cancer

Dong-Xu He, Feng Gu, Fei Gao, Jun-jun Hao, Desheng Gong, Xiao-Ting Gu, Ai-Qin Mao, Jian Jin, Li Fu, Xin Ma

**Supplemental materials and methods**

**The single-enzyme RRBS library preparation**

RRBS libraries with single MspI digestion were constructed for MCF-7/WT, ADM and PTX cell line, as previously described^1^. Briefly, 100 ng of genomic DNA was digested with 300U of MspI enzymes (New England Biolabs Ltd., Hitchin, UK) in 100 μl reactions at 37°C for 16–19 h. After purification, the digested products were blunt-ended, and then dA was added, followed by methylated-adapter ligation. To obtain DNA fractions of 40-120 bp and 120-220 bp ranges of MspIdigested products, two ranges of 160-240 bp and 240-340 bp adapter-ligated fractions were excised from a 2% agarose gel, respectively. Bisulfite conversion was conducted using a ZYMO EZ DNA Methylation-Gold Kit™ (Zymo Research, CA, USA), following the manufacturer’s instructions. The final libraries were generated by PCR amplification using JumpStart™ Taq DNA Polymerase (Sigma, USA). RRBS libraries were analyzed by an Agilent 2100 Bioanalyzer (Agilent Technologies, CA, USA) and quantified by real time PCR.

**RRBS sequencing and data analysis**

The RRBS libraries were sequenced using Illumina Hiseq2000 according to the manufacturer’s instructions (Illumina Inc., CA, USA). Raw sequencing data was processed by the Illumina base-calling pipeline. Low quality reads that contained more than 30% ‘N’s or over 10% of the sequence with low quality value (quality value < 20) per read were omitted from the data analysis. The clean bisulfite sequencing reads were aligned to the UCSC human reference genome (hg19, <http://hgdownload.soe.ucsc.edu/goldenPath/hg19/>) with an unbiased three-letter (namely, A, G and T) way which converting all Cs into Ts in the reads and for both strands of the genomic DNA sequence. The SOAPaligner (<http://soap.genomics.org.cn/index.html> ) was used for sequencing alignment, and then the uniquely mapped reads without duplication were kept for further analysis. Methylation levels of cytosines were analyzed as previously described^1^. DMRs (Differential Methylated Regions) were detected pairwised by scanning methylation level of the whole genomic CpG sites^2^. For each of DMRs, the number of methylated and unmethylated CpG reads was counted, a chi square test was applied to identify differentially methylated genomic elements with a threshold of p-value < 0.01. Meanwhile, the difference in methylation levels between two samples should be more than 20%. Promoters were defined as the regions spanning 2200 bp upstream and 500 bp down stream of the transcriptional start site.

**RNA-seq and statistical analysis**

The total RNA samples were first treated with DNase I to degrade any possible DNA contamination. Then the mRNA was enriched by using the oligo (dT) magnetic beads. Mixed with the fragmentation buffer, the mRNA was fragmented into short fragments (about 200 bp). Then the first strand of cDNA was synthesized by using random hexamer-primer. Buffer, dNTPs, RNase H and DNA polymerase I were added to synthesize the second strand. The double strand cDNA was purified with magnetic beads. End reparation and 3’-end single nucleotide A (adenine) addition was then performed. Finally, sequencing adaptors were ligated to the fragments. The fragments were enriched by PCR amplification. During the QC step, Agilent2100 Bioanaylzer (Agilent Technologies, CA, USA) and ABI StepOnePlus Real-Time PCR System (Applied Biosystems, Foster City, USA) were used to qualify and quantify of the sample library. The library products were ready for sequencing via Illumina HiSeq2000.

The following data processing including gene expression level and differentially expressed genes were statistically analysed by the method of Audic and Claverie^3^. mRNA abundance was determined by RPKM index^4^ (Reads Per kb per Million reads). If there was more than one transcript for one gene, the longest one was used to calculate its expression level and coverage. The RPKM values can be directly used for comparing the difference of gene expression among samples. The significantly differentially expressed genes (DGE) were determined at a threshold false discovery rate (FDR) ≤0.001 and the absolute value of log2Ratio ≥ 1.

**Small RNA-seq and data analysis**

The 50bp single-end sequence reads from Illumina HiSeq2000 sequencing were performed through the data cleaning first, which includes getting rid of the low quality reads and several kinds of contaminants from the 50bp reads. Afterwards, the standard bioinformatics analysis was used to annotate the clean reads into different categories small RNA, and reads which could not be annotated to any category were used for the novel miRNA prediction and base edit of potential known miRNA. The SOAPaligner was used for sequencing alignment, and the uniquely mapped reads were kept for further analysis. The miRBase18 (<ftp://mirbase.org/pub/mirbase/18/>) was used to annotate the uniquely mapped small RNA sequences. Known miRNA expression profiles and differential miRNA expression were calculated by same method and threshold in RNA-seq.

**Graphics and heatmaps**

All graphs were created using R (<http://cran.rstudio.com/>) and perl module SVG (<https://github.com/szabgab/SVG>). Heatmaps were generated from the expression or methylation data using R package ‘gplots’.

Network analysis

molecular interactions were analyzed by Cytoscape_3.2.0^5^ plugin GeneMANIA^6^ based on BioGRID^7^ datasets of H.sapiens (human). The query genes were marked blue, and the genes in 17-gene signature were marked yellow.

**Reverse transcription (RT)-PCR**

Total RNA was isolated from confluent monolayers of MCF-7 cells by Trizol (Invitrogen, CA, USA) and cDNA was synthesized from total RNA (3µg) by using the SuperScript First-Strand Synthesis System (Invitrogen, CA, USA) with Oligo (dT) primers. The cDNA obtained was used to run PCR with pairs of primers.  To normalize mRNA expression levels, β-actin was used as an endogenous internal control. Primers used for RT-PCR of E-cadherin, N-cadherin, Vimentin, Snail1, Twist1 were as follows:  β-actin forward, 5’- tgtcaccaactgggacgata-3’ and reverse 5’- tctcagctgtggtggtgaag-3’; E-cadherin-forward*, 5’-gctggagattaatccggaca-3’* and reverse*, 5’-* *acccacctctaaggccatct-3’;* N-cadherin-forward*, 5’-* *acagtggccacctacaaagg-3’* and reverse*, 5’-* *tgatccctcaggaactgtcc-3’;* Vimentin-forward*, 5’-* *ggctcagattcaggaacagc-3’* and reverse*, 5’-* *gcttcaacggcaaagttctc-3’;* Snail1-forward*, 5’-* *tgtctgcgtgggtttttgta-3’* and reverse*, 5’-* *ccgacaagtgacagccatta-3’;* Twist1-forward*, 5’-* *tcctctaccaggtcctcca-3’* and reverse*, 5’- cacgccctgtttctttgaat-3’.*PCR was executed for 35 cycles of 95˚C for 30 sec, 58˚C for 30 sec and 72˚C for 45 sec. RT-PCR products were separated through a agarose gel (1% w/v) and analyzed with ImageJ software (National Institutes of Health, MD, USA).

**qRT-PCR**

miRNA expression was analysis by qRT-PCR with All-in-One™ miRNA qRT-PCR Detection Kit(GeneCopoeia, CA, USA). Briefly, total RNA was extracted in MCF-7/WT and ADM cells with Trizol (invitrogen, CA, USA). Then mature miRNAs were reverse transcribed in the presenceof a poly-A polymerase with an oligo-dT adaptor. qRT-PCR was then performed with miRNA primers (GeneCopoeia, CA, USA) at 60℃ for 20s, 72℃ for 10s, 40 cycles with iQ5 Real Time PCR Detection System (Bio-Rad, CA, USA), the expression of miRNAs were normalizedto the expression of U6 small RNA.

**Migration assay**

The transwell migration assays was performed with the modified Boyden chamber method in 24-well plates, each containing 6.5-mm Transwell chambers with 8-mm pores (BD Biosciences, CA, USA). The medium in the wells of chamber contained 10% fetal calf serum as a source of chemoattractant. For seeding, Cells were harvested, washed twice with PBS and resuspended in serum-free RPMI-1640. 1×10^5^ cells for MCF-7/WT, MCF-7/ADM and MCF-7/PTX were seeded into the upper chamber of each Transwell unit. After 24 h, the cells that had not migrated through the membrane in the Transwell inserts (on the upper surface) were removed with a cotton swab. Cells that had passed through the membrane were stained with crystal violet and counted under a microscope. Images were captured by a video camera (Nikon Coolpix 54,  Nikon Corporation, Tokyo, Japan) mounted on the microscope (Leica CME, Leica Microsystems, Wetzlar, Germany). Cell migration was quantified by direct microscopic visualization and counting. The values for migration was considered as the mean number of migrated cells by counting four fields per membrane and the results are presented as the average of at least three independent experiments performed.

**Immunofluorescence staining**

The cells were seeded into confocal dishes and left overnight. After 24h, the cells were washed with PBS and fixed for 30 min in 4% paraformaldehyde, permeabilized with 0.1% Triton X-100 for 10 min at room temperature, respectively. Cells were washed 3 times in PBS after each treatment. They were then blocked with 2% BSA in PBS for 30 min at room temperature, incubated overnight at 4˚C with primary antibodies diluted in 2% BSA/PBS. After the incubation time, cells were washed three times for 5 minutes with PBS, then incubated with secondary antibodies for 1 h at room temperature with donkey anti rabbit Alexa 568 and donkey anti goat Alexa 546(1:200, Invitrogen, CA, USA) diluted in 2% BSA/PBS. After washing again with PBS, the nuclei were counterstained with 10 μg/mL DAPI for 10 min at room temperature to observe the number of cell nucleis. The stained samples images were acquired with a confocal laser scanning microscope (LSM 710; Carl Zeiss, Jena, Germany).

**Detection of IC_50_**

The MCF-7 cells were transfected by miRNA mimics or inhibitors and seeded in the 96-well plate, then the cells were treated with 2-fold serially diluted ADM or PTX for 48h. MTT assay were then applied to detect the changes in cell viability, the IC_50_ were calculated by nonlinear regression in Prism Graphpad software.

**Ethics statement**

Each patient involved in this study had provided written informed consent. All studies in enrolling patients or in sample analysis were conducted with approval from the ethics committees of the university and hospital.

**Immunohistochemistry Staining**

Tissue slides were deparaffinized and blocked for endogenous peroxidase activity. Primary antibodies were incubated at 4 °C. Subsequently, the sections were incubated with the GTVision III Detection System/Mo&Rb Kit (Gene Tech Co., Ltd., Shanghai, PR China).

All staining was assessed by pathologists blinded to the origination of the samples and subject outcome. Each specimen was assigned a score according to the intensity of the membrane staining (0, no staining; 1, weak staining; 2, moderate staining; 3, strong staining).

**GEO database validation**

The raw data of GEO databases were downloaded from NCBI website. Data for Cy3 and Cy5 channels were Lowess-normalized and then log(2) ratio was taken. Multiple probe sets for the same transcript were averaged after excluding non-specific probe sets that ended with “_x_at”^8^. The Bayesian binary regression, k-nearest neighbor and Kaplan-Meier methods were analyzed in SPSS modeler software^9-11^.

**Supplemental figures**


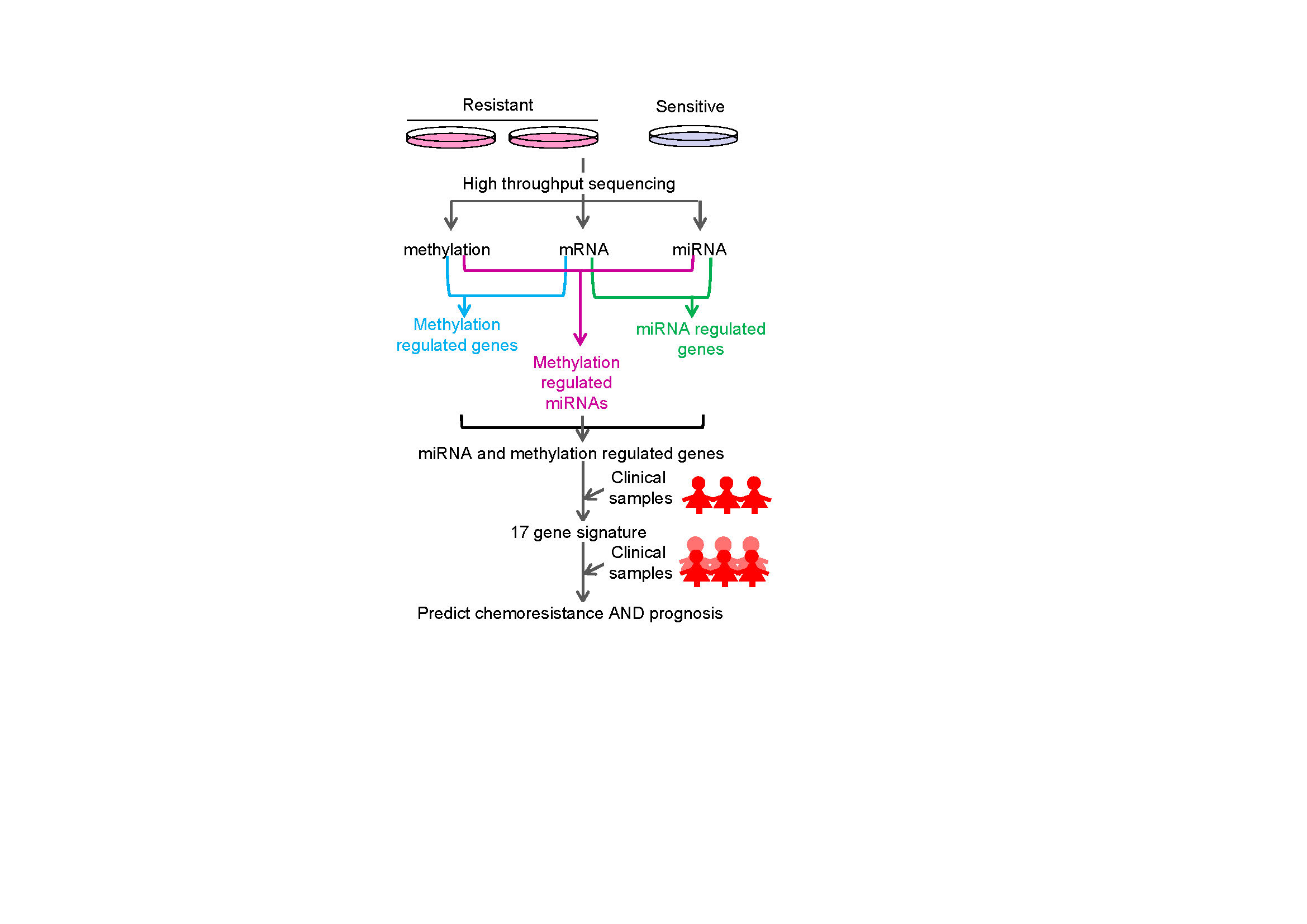


Fig S1. Work flow of this study. Chemoresistant and sensitive breast cancer cells were analyzed by RRBS, RNA-Seq. The sequencing data were analyzed by cross matching with each other. Based on the analysis, a chemoresistance signature was generated and validated in clinical samples.

**
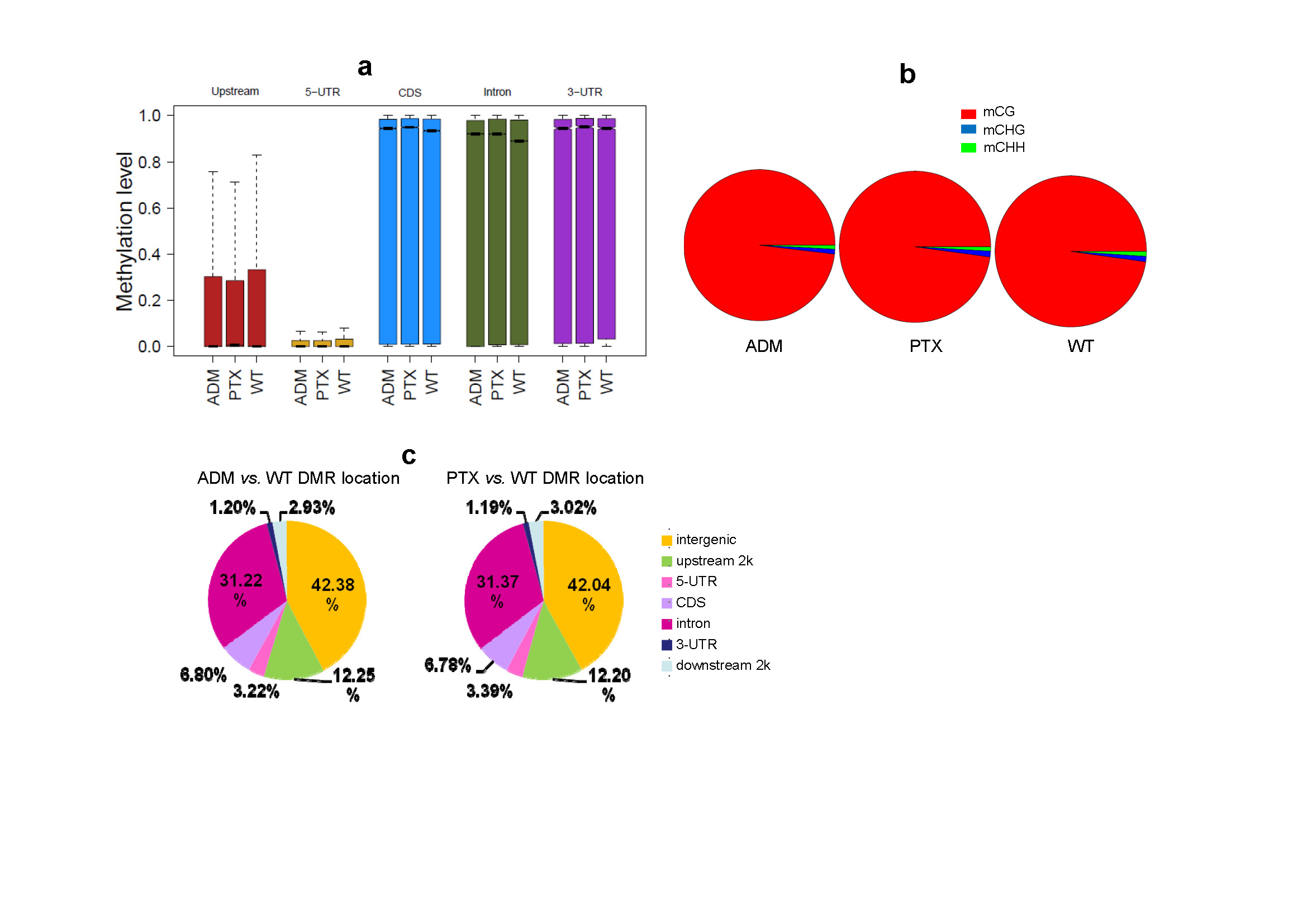
**

**Figure S2** (a) methylation levels in coding sequences, introns, 3’-untranslated regions (UTRs), 5’-UTRs and promoters in MCF-7/ADM, /PTX and /WT cells. (b) methylated cytosines distribution in CpG, CHG and CHH contexts. (c) DMRs location in gene-body, intergenic regions, promoters, 3’ and 5’-UTR regions.


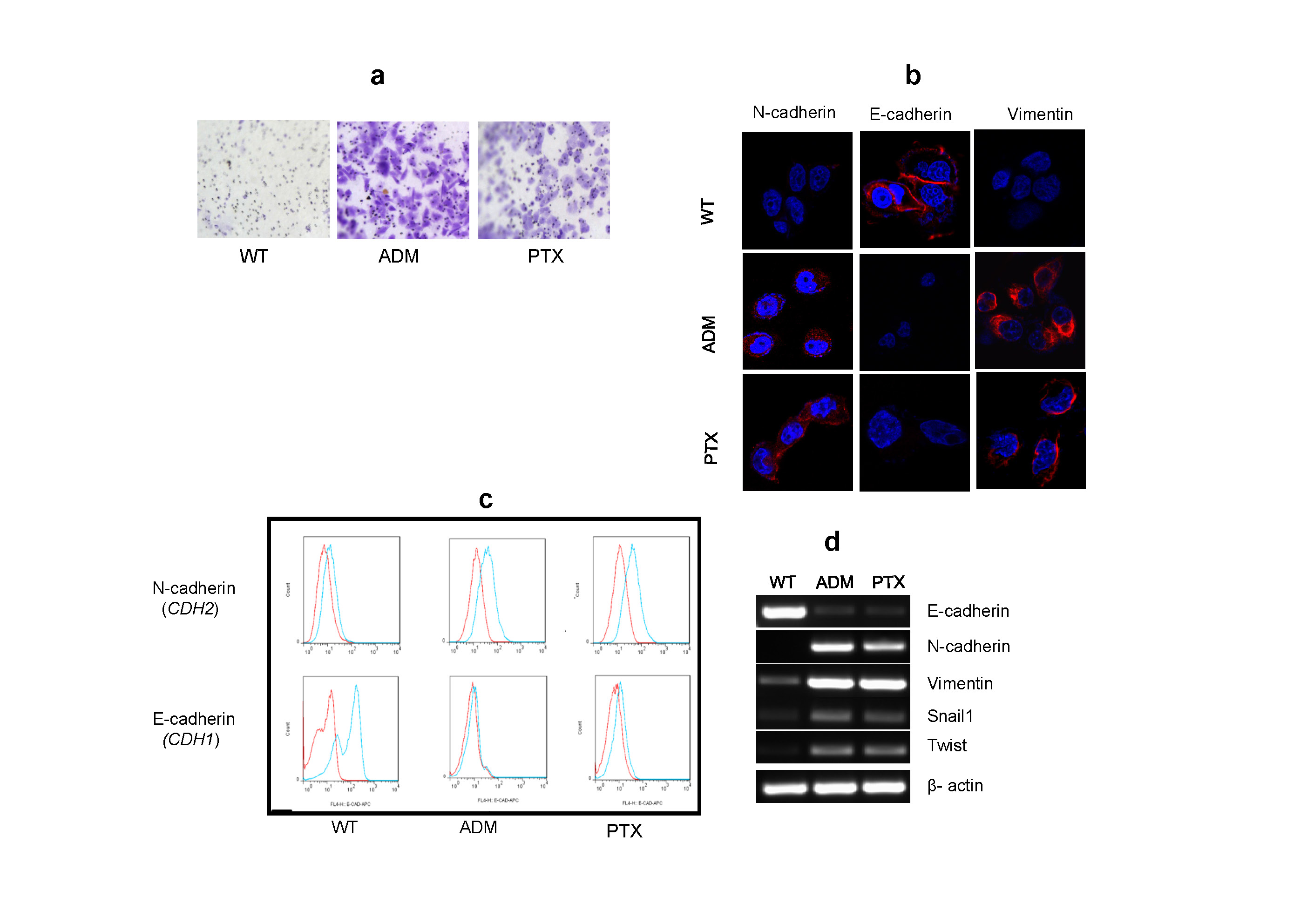


**Figure S3** EMT status of chemoresistant cells. (a) The cell motility was increased in MCF-7/ADM and PTX cells versus WT cells. (b-d) Expression of N-cadherin (*CDH2*)， E-cadherin(*CDH1*), vimentin (*VIM*), snai1, twist genes/ proteins were analyzed by Immunofluorescence staining, flow cytometry and RT-PCR.


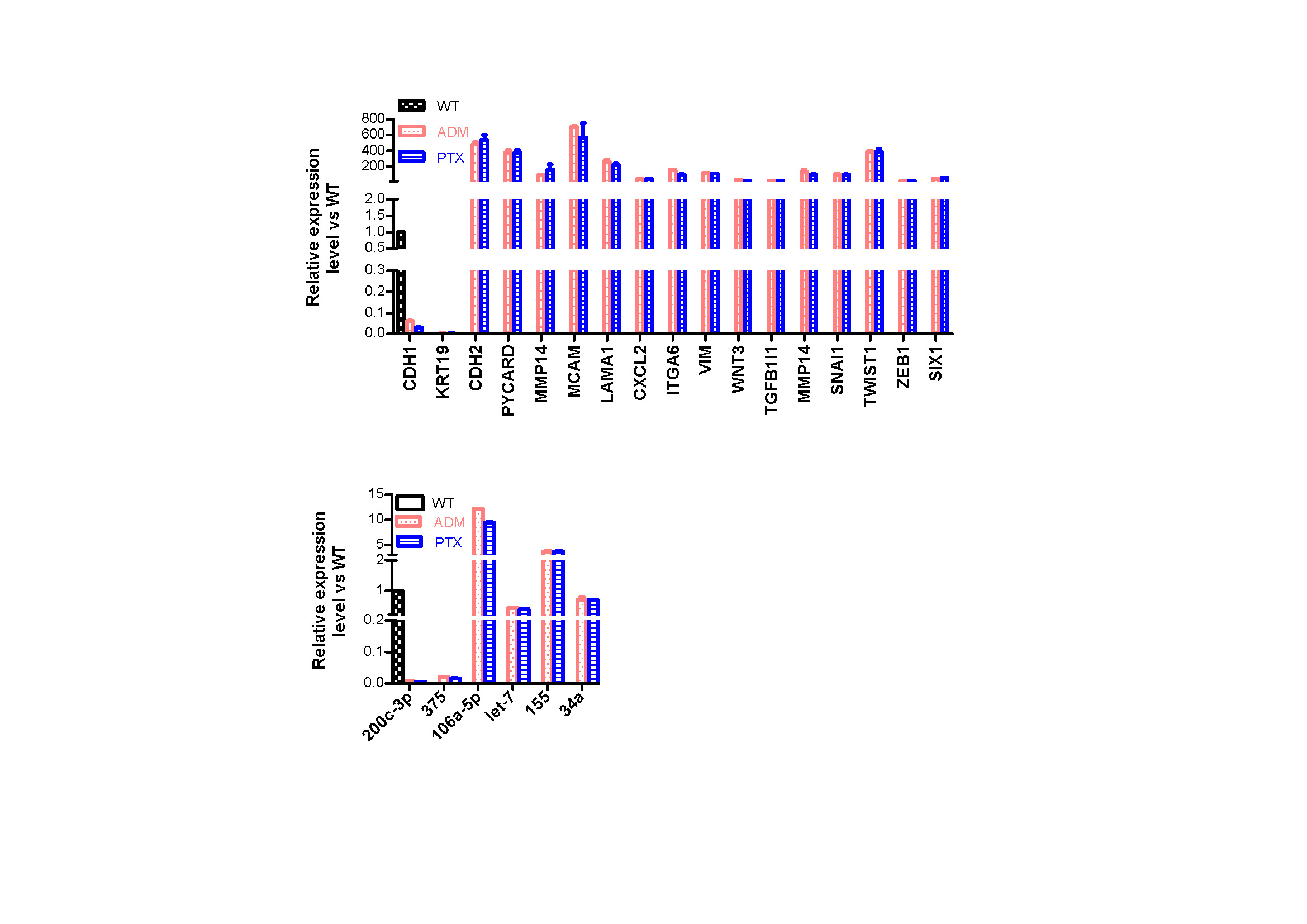


**Figure S4** mRNA level of several EMT-related genes and miRNAs, which was analyzed by real-time PCR.


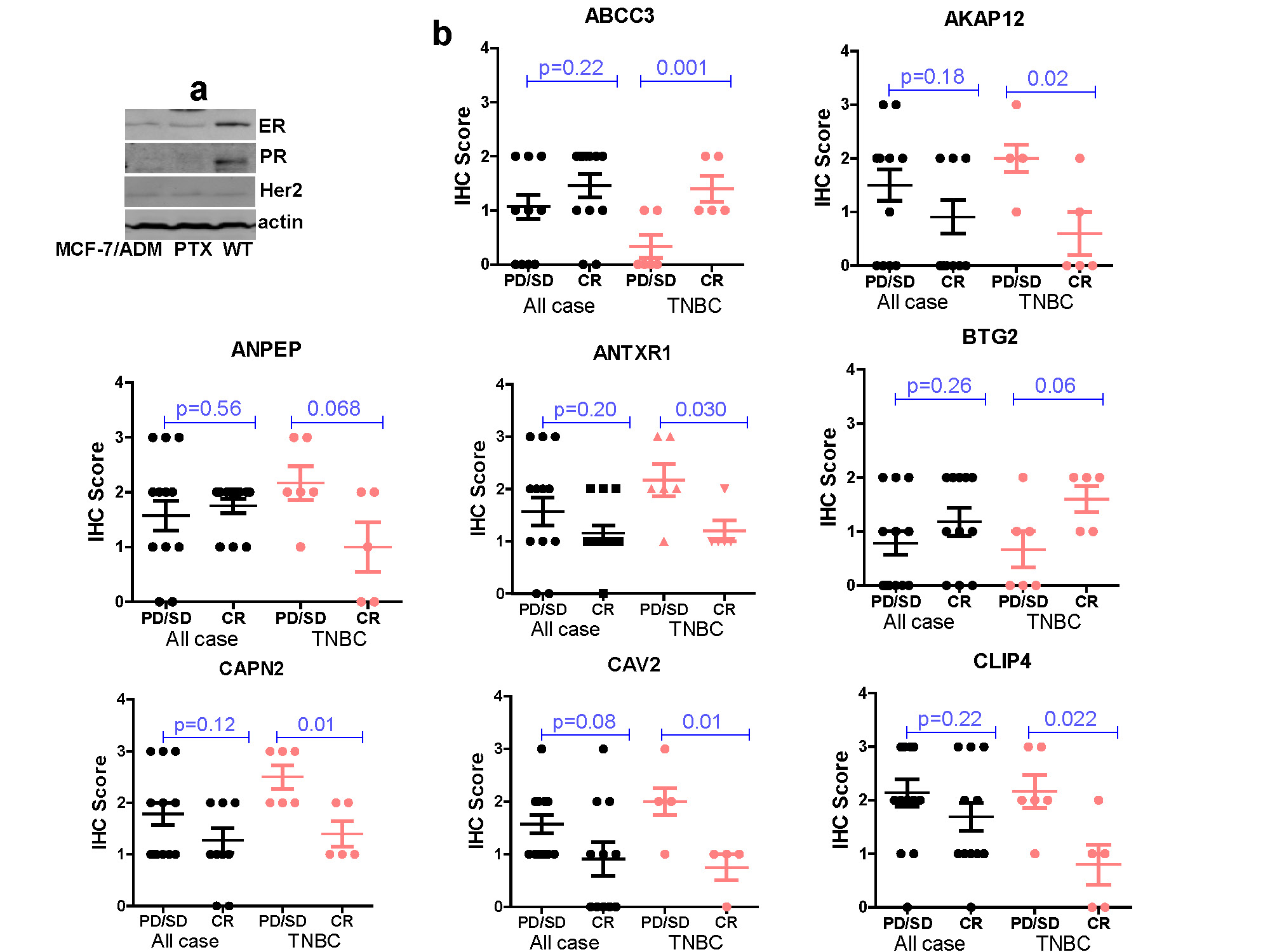


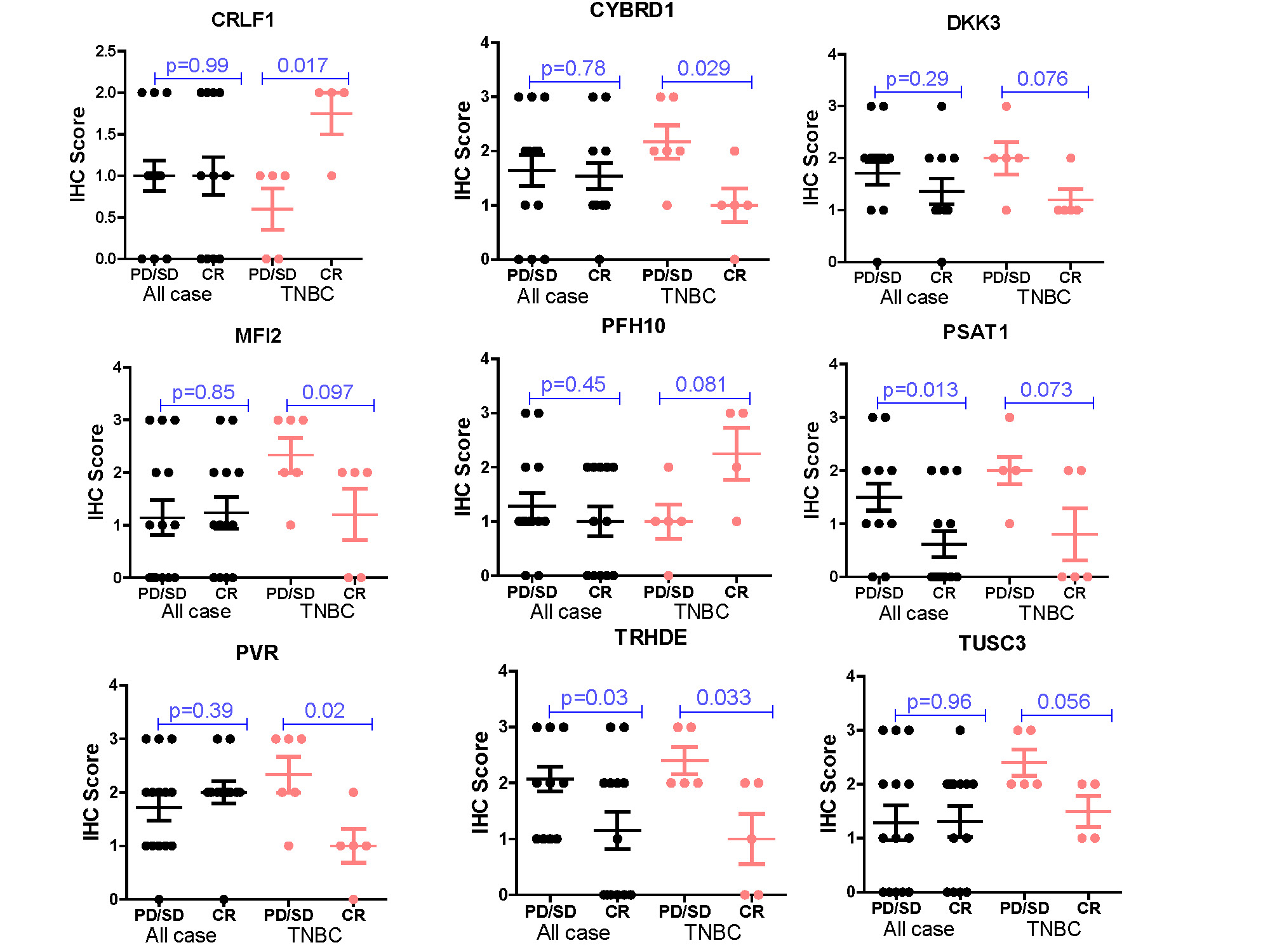


**Figure S5** Validation the signature in clinical samples. (a) the ER, PR and Her2 status of MCF-7/ADM and PTX cells and WT cells. (b) Immunohistochemical analysis of the genes in the signature.

References

1. Gu H, Bock C, Mikkelsen TS, Jager N, Smith ZD, Tomazou E, Gnirke A, Lander ES, and Meissner A (2010) Genome-scale DNA methylation mapping of clinical samples at single-nucleotide resolution. Nat Methods 7 (2):133-136

2. Wang J, Xia Y, Li L, Gong D, Yao Y, Luo H, Lu H, Yi N, Wu H, Zhang X, Tao Q, and Gao F (2013) Double restriction-enzyme digestion improves the coverage and accuracy of genome-wide CpG methylation profiling by reduced representation bisulfite sequencing. BMC Genomics 14:11

3. Audic S and Claverie JM (1997) The significance of digital gene expression profiles. Genome Res 7 (10):986-995

4. Mortazavi A, Williams BA, McCue K, Schaeffer L, and Wold B (2008) Mapping and quantifying mammalian transcriptomes by RNA-Seq. Nat Methods 5 (7):621-628

5. Shannon P, Markiel A, Ozier O, Baliga NS, Wang JT, Ramage D, Amin N, Schwikowski B, and Ideker T (2003) Cytoscape: a software environment for integrated models of biomolecular interaction networks. Genome Res 13 (11):2498-2504

6. Montojo J, Zuberi K, Rodriguez H, Kazi F, Wright G, Donaldson SL, Morris Q, and Bader GD (2010) GeneMANIA Cytoscape plugin: fast gene function predictions on the desktop. Bioinformatics 26 (22):2927-2928

7. Chatr-Aryamontri A, Breitkreutz BJ, Oughtred R, Boucher L, Heinicke S, Chen D, Stark C, Breitkreutz A, Kolas N, O'Donnell L, Reguly T, Nixon J, Ramage L, Winter A, Sellam A, Chang C, Hirschman J, Theesfeld C, Rust J, Livstone MS, Dolinski K, and Tyers M (2015) The BioGRID interaction database: 2015 update. Nucleic Acids Res 43 (Database issue):D470-D478

8. Liu M, Liberzon A, Kong SW, Lai WR, Park PJ, Kohane IS, and Kasif S (2007) Network-based analysis of affected biological processes in type 2 diabetes models. PLoS Genet 3 (6):e96

9. Li Q, Birkbak NJ, Gyorffy B, Szallasi Z, and Eklund AC (2011) Jetset: selecting the optimal microarray probe set to represent a gene. BMC Bioinformatics 12:474

10. Liu M, Liberzon A, Kong SW, Lai WR, Park PJ, Kohane IS, and Kasif S (2007) Network-based analysis of affected biological processes in type 2 diabetes models. PLoS Genet 3 (6):e96

11. Elbez Y, Farkash-Amar S, and Simon I (2006) An analysis of intra array repeats: the good, the bad and the non informative. BMC Genomics 7:136
